# College and Hospital Notes

**Published:** 1907-09

**Authors:** 


### COLLEGE AND HOSPITAL NOTES.

At the German Hospital, Buffalo, a memorial to Gerhard Lang was unveiled August 15, 1907. This consisted of a bronze tablet commemorating the philanthropies of Mr. Lang, who had been especially generous to the hospital, and who died about fifteen years ago. Dr. C. H. W. Auel, vice-president of the hospital, presided at the unveiling ceremonies, introducing Jacob Stern, one of the directors, who pronounced the formal eulogy. After referring to Mr. Lang's work in and for Buffalo the speaker among other things said:

"Mr. Lang was always interested in charitable undertakings and especially hospital work. When the German Hospital was organised his children gave the property upon which this hospital now stands. Tonight we dedicate this tablet to the memory of Mr. Lang and I now ask you, Mr. Kasting, as president of the hospital, to accept the same."

President Kasting then made a short address. He paid a tribute to Mr. Lang and to William Simon, former president of the hospital, who, he said, had done much to place it on its present sound financial basis; also to the Ladies' Sewing Circle, which has taken a deep interest in the hospital. Ottomar Reinecke then spoke briefly in German.

Neither the sculptor, A. A. Langenbahn nor Dr. Herbert Rust, former editor of the Freie Presse, who wrote the inscription for it, lived to see the tablet placed in the hall of the hospital. Both have died within the past few months.

---

The University of Buffalo, medical department, will begin its sixty-second regular session Monday, September 23, 1907, and continue thirty-four weeks. This is one of the earlier colleges to lengthen its term beyond the legal requirements. Dr. Allen A. Jones, adjunct professor of the principles and practice of medicine, will deliver the opening lecture at the time above specified.

---

The United States Marine Hospital, in Buffalo, now seems to be an assured fact. It will be remembered that an appropriation was made by congress for this purpose some years ago; but for one reason and another there has been delay in realising the money. Finally, however, the supervising architect at Washington has sent on to Buffalo plans and specifications. Sealed proposals for doing the work are to be sent to Washington and will be received there not later than September 16, 1907. The site for the hospital is on Main street between Dewey avenue and Humboldt parkway.

---

A Polish hospital is projected in Buffalo and it is expected that on or about September 1, ground will be broken for the building, which is to occupy a whole block included between Fillmore avenue, Stanislaus, Beck and Gibson streets. The hospital when completed will cost $500,000 or more and will be built entirely at the expense of the Polish people of Buffalo. Two priests and a layman, whose names are not public, recently gave money sufficient to buy a lot, 205 feet front, on Stanislaus street, and having a depth of 297 feet. An option has been obtained upon the rest of the block and the purchase will be consummated soon.

The hospital will be in charge of the Polish Sisters of Charity and upon the board of trustees will be a representative from each of the ten parishes in the Polish district.

Dr. Francis E. Fronczak, assistant health commissioner, is one of the projectors deeply interested in the enterprise.

---

THE College of Medicine, Syracuse University, has a four years' course of study. Its facilities for instruction are unusually good. They consist of a main building erected in 1896, containing a library of several thousand volumes, study and lecture rooms, and laboratories large, light, convenient and well equipped with the apparatus required in the study of the fundamental medical sciences. Its facilities also include access to three large hospitals and three institutions for purposes of clinical instruction.

